# Impact of Clinical Experience and Diagnostic Performance in Patients with Acute Abdominal Pain

**DOI:** 10.1155/2015/590346

**Published:** 2015-01-22

**Authors:** Helena Laurell, Lars-Erik Hansson, Ulf Gunnarsson

**Affiliations:** ^1^Department of Surgery, Mora Hospital, 792 51 Mora, Sweden; ^2^Department of Surgery, Sahlgrenska University Hospital, 413 46 Gothenburg, Sweden; ^3^Department of Surgical and Perioperative Sciences, Umeå University, 901 95 Umeå, Sweden

## Abstract

*Background.* The aims were to evaluate the importance of the formal competence of the emergency department physician, the patient's time of arrival at the emergency department, and the use of a structured schedule for investigation of patients with acute abdominal pain. *Methods.* Patients attending the Mora Hospital with acute abdominal pain from 1997 to 2000 were registered prospectively according to a structured schedule. Registration included history, symptoms, signs, preliminary diagnosis, surgery and final diagnosis after at least one year.  *Results.* 3073 acute abdominal pain patients were included. The preliminary diagnosis, as compared with the final diagnosis, was correct in 54% (*n* = 1659). Previously, during 1996, a base-line registration of 790 patients had a 58% correct diagnoses  (*n* = 458). A majority of the patients (*n* = 2699; 88%) were managed by nonspecialists. The proportion of correct diagnoses was 54% (*n* = 759) for pre-registrar house officers and 55% (*n* = 443) for senior house officers. Diagnostic performance at the emergency department was independent of patient's time of arrival. *Conclusions.* A structured schedule for investigation did not improve the diagnostic precision at the emergency department in patients with acute abdominal pain. The diagnostic performance was independent of the formal competence of the physician and the patient's time of arrival.

## 1. Background

Acute abdominal pain emanates from a spectrum of aetiologies, some of which are severe, but in the majority of cases the reason for the pain cannot be determined. Thus, it will be classified as nonspecific abdominal pain (NSAP). Acute abdominal pain accounts for a substantial proportion of patients arriving at a surgical emergency department [1–6]. Many of the patients with NSAP are hospitalised for observation only and the cost to society of admissions for observation is considerable [7–9]. On the contrary, a small proportion of the patients arriving at a surgical emergency department with abdominal pain regain urgent intervention with surgery or other advanced treatment [3, 6, 10]. Thus, to make the most correct diagnosis possible is important for both patient safety and health economy.

Previous investigations have revealed a proportion of correct diagnoses in acute abdominal pain ranging from 40 to 73% [8, 11–16]. Algorithms for standard use in the emergency department [8, 14, 16–18], mathematical models [1, 14, 18, 19], standardised schedules for recording history, symptoms, signs, and computer-aided diagnostics have all been tried with the aim of improving the diagnostic performance [1, 8, 11, 12, 14, 16, 20, 21]. With such methods a diagnostic performance of up to 91.8% has been reached [1]. In recent years, imaging [22–24] and laparoscopy [25] have been suited in the perspective of effectiveness and efficacy in diagnostics of emergency abdominal pain with diverging outcomes. Few studies [26, 27], however, have focused on the formal competence of the emergency physician as a determining factor. Our aim with the present study was to investigate the precision of the preliminary diagnosis at the emergency department as made by on-call physicians classified according to their degree of experience. Furthermore, we introduced a detailed schedule for recording the history, symptoms, clinical signs, and results of laboratory investigations to allow analysis of possible effects on the diagnostic performance. The precision of diagnosis was also classified in relation to the patient's time of arrival at the emergency department.

## 2. Methods

Mora Hospital in the county of Dalarna, Sweden, is a district hospital with a catchment population of about 87,000. The hospital provides full 24 h emergency service with surgery, X-ray, an intensive care unit, and on-call consultants also cover gynaecology and internal medicine. At the time of the study, at the department of surgery, there was one physician on call, most often undergoing continuing education but with an experienced consultant available at a few minutes' notice. Categories of attending physicians are listed in Table 1. The most junior physicians are the locums with 0 to 2 years of medical experience, pre-registrar house officers with 0.5 to 1 year experience of clinical practice after university medical qualification, followed by the senior house officers with 1–5 years' experience of surgery. During night-time, some general physicians from the primary health care services also participated as on-call physicians at the emergency department. The specialists and consultants concerned in this study were generally well experienced with many years in the profession.

Patients admitted with abdominal pain of up to seven days' duration were included in the study. A baseline registration of logistic data such as time of admission, the level of formal competence of the attending physician and the preliminary diagnosis was performed during the period February 1, 1996, to January 31, 1997. During the subsequent study period, February 1, 1997, to June 1, 2000, the initial formulary was supplemented with a detailed schedule for history, symptoms, clinical signs, and results of laboratory investigations. In the latter formulary the physician was asked to give a first and a second most possible diagnosis, whereas during the baseline registration period only the most possible diagnosis was registered.

### 2.1. Patients Included

Inclusion criterions were age above one year and acute abdominal pain of up to seven days' duration, not caused by trauma. On the basis of these criteria, 12 of the 3349 patients registered in the database were excluded. The included patients (*n* = 3337) were divided into two groups, those living within the hospital catchment area and those who were not (Table 2). The reason was that only the former group was to be included in population-based analyses, and only for this group were follow-up data accessible in the hospitals records. During the study period with the detailed schedule, corresponding data were also submitted from three primary health care centres within the hospital catchment area.

Out of the 3337 patients included (Table 2), 2979 (89%) were living within the hospital catchment area and were eligible for follow-up.

In calculations of the proportion of correct preliminary diagnosis, however, patients living outside the hospital catchment area were also included if the final diagnosis was considered certain (i.e., the patient was operated on with a conclusive finding). According to this criterion, 3073 records (92%) were eligible for calculation. The primary health care centres contributed with 238 patients, of whom 222 (93%) were eligible for follow-up. Corresponding number for the baseline registration year was 790 out of 881 (90%).

### 2.2. Computer Registration

When arriving at the emergency department, all patients who fulfilled the inclusion criteria had the study formulary included in their medical record. The attending physician registered data for history, symptoms, clinical signs, and preliminary diagnosis before the patient left for admission to a ward or was discharged. Furthermore, data for results of laboratory investigations, surgery, duration of hospitalisation, and diagnosis at discharge were registered by the physician responsible for that decision. All formularies were checked by a specially trained secretary and entered into a Microsoft Access database. At this time, any obviously erroneous information detected was corrected and at computer registration logical filters detecting impossible or inconsistent combinations of data were applied.

### 2.3. Follow-Up Data

Records of all patients residing within the hospital catchment area were checked at least one year (mean 2.7 years) after admission. Follow-up was performed by checking the patient's record at the surgical department and the primary health care centre, and if necessary also records from other departments at the hospital. Further investigations were registered and the discharge diagnosis was reevaluated according to criteria of the World Organisation of Gastroenterology [28]. This reevaluated, final diagnosis served as the basis for calculations of the reliability of the preliminary diagnosis registered on admission to the emergency department and the diagnosis at discharge.

### 2.4. Validation

Before any calculations were performed, all stochastic or continuous variables were cleared of evidently erroneous information in that all data out of the 75th percentile were checked against record data. As a check of the validity of registration, 300 cases (10%) were randomised for validation. Data for those cases were checked against the medical records and if necessary against the hospital computer system for time of arrival, time of surgery, and so forth. All erroneous data detected in these two steps were corrected in the register. Results of the validity check are given in Table 3.

The completeness of the registration was first checked by the secretaries, who indicated cases discharged with a history of acute abdominal pain without register formularies included in their medical records. In such cases the physicians responsible for the present study checked if the case was eligible for the study, and if so, diagnosis at discharge, surgery, and laboratory parameters were registered. During this procedure another 523 patients who should have been included in the database were found.

At the emergency department, all patients admitted are routinely registered by the nurses according to type of symptoms and signs. Those registrations were scanned for symptoms possibly related to acute abdominal pain, on randomised days of admission. Patients with a symptom related to abdominal pain were checked against the study register and if not present, the medical records were checked for possible fall-off. With this method the overall completeness was calculated to be 79%.

### 2.5. Statistical Methods

Statistics were calculated by the Statistica software (Statsoft, Tulsa, USA). The distribution for continuous and stochastic variables was considered normal distribution as judged by the Kolmogorow-Smirnow test for the entire population. However, when calculations were made on smaller samples, some parameters did not fit in that distribution model and thus nonparametric statistics were used. Differences between groups were calculated by the Mann-Whitney* U* test and if dichotomous, by the Chi-square test. The proportion of correct preliminary diagnosis was calculated against the final diagnosis after follow-up.

## 3. Results

**Figure 1:**
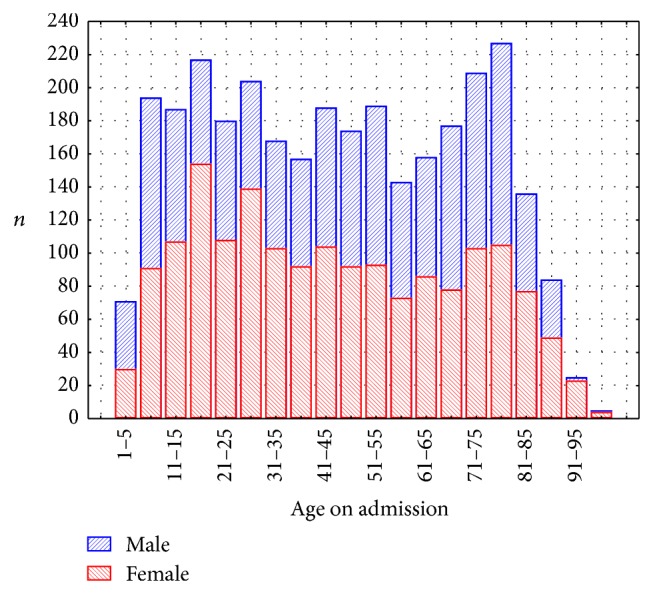
Age and gender distribution.

The mean age of patients admitted to hospital with acute abdominal pain was 46 years (Table 2), with a male/female ratio of 0.82 (*n* = 1382 : 1691). The age distribution is shown in Figure 1, where it is seen that the curve is binomial, with two peaks, at 20 and 75 years, respectively. At the ages 15 to 45 years and over 90 years the majority of patients were females.

Of the 2851 patients admitted to the emergency department, 72% (*n* = 2062) were treated as in-patients whereas 789 (28%) were treated as out-patients.

### 3.1. Distribution of Diagnoses and Sensitivity

The distributions of the preliminary diagnoses made on admission to the emergency department, of the final diagnoses at follow-up, and of the preliminary diagnoses made at the primary health care centres are listed in Table 4. As seen in the table, the ten most common final diagnoses at the emergency department were NSAP (37%, *n* = 1058), gallbladder disease (10.5%, *n* = 334), appendicitis (9.8%, *n* = 277), diverticulitis (4.7%, *n* = 134), constipation (4.6%, *n* = 130), ureteric stone (4%, *n* = 107), gynaecological complaints (3.5%, *n* = 101), acute pancreatitis (3.2%, *n* = 92), acute intestinal obstruction (3.2% *n* = 92), and urinary tract infection (2.6%, *n* = 74). The total rate of detected abdominal malignancies was 2.8% (*n* = 86). The sensitivities for the preliminary diagnoses at the emergency department were as follows: appendicitis 0.80, cholecystitis 0.51, gallstones 0.68, diverticulitis 0.64, and ureteric stone 0.78.

### 3.2. Diagnostic Performance

A majority of the patients attending the emergency department (88%, *n* = 2699) were managed by a nonspecialist physician with 0.5 to 5 years of experience. Most patients, 46% (*n* = 1409), were seen by pre-registrar house officers, 16% (*n* = 479) by locums and 26% (*n* = 811) by senior house officers, with proportions of correct diagnoses of 54 (*n* = 759), 58 (*n* = 277) and 55% (*n* = 443), respectively (Table 1). There was no difference in diagnostic performance according to category of physician. During the baseline period the pre-registrar house officers were in contact with the consultant in 34% (*n* = 143) of the cases, whereas the senior house officers had such contact in only 12% (*n* = 41) of the cases (*P* < 0.001). A general physician on duty at the emergency department during the night-time handled 6% (*n* = 195) of the patients, with a rate of 53% (*n* = 103) correct diagnoses. For patients attending primary health care centres, the rate of correct diagnoses was 51% (*n* = 71). The diagnostic performance was higher for out-patients both during the baseline (65%, *n* = 149) and study period (63%, *n* = 694). The accuracy rate for the preliminary diagnosis at the emergency department was lower (*P* < 0.001) for women (52%, *n* = 1691) than in men (58%, *n* = 1382). The diagnostic performance at the emergency department was independent of the patient's time of arrival. The rate of correct diagnoses from midnight to 6 a.m. was 52% (*n* = 434), from 3 a.m. to 6 a.m. 52% (*n* = 190), and from 6 a.m. to midnight 55% (*n* = 2639). During the study period, the precision of the preliminary diagnoses increased with time, 51% (*n* = 494; *n* = 968) during 1997, 54% (*n* = 585; *n* = 1090) during 1998, 55% (*n* = 418; *n* = 764) during 1999, and 57% (*n* = 143; *n* = 251) during 2000. When the second preliminary diagnoses was also included in the calculation of correct diagnoses, the overall accuracy rate increased to 58% (*n* = 558) during 1997, 62% (*n* = 680) during 1998, 61% (*n* = 468) during 1999, and 60% (*n* = 151) during 2000.

## 4. Discussion

A structured schedule for history and clinical examination has been advocated in previous studies [13, 15, 16]. In those studies the introduction of such a schedule improved the diagnostic performance by 5–20% [12, 13, 15, 20]. In our study, however, the introduction of a schedule for investigation did not improve the diagnostic performance as compared to that in the baseline registration year. Nevertheless the proportion of correct diagnoses increased each year, although no education or feedback was given to the physicians as in some of the other studies [8, 12, 13, 16].

During the study period 1997 to 2000, the physicians were given the opportunity to add a second alternative diagnosis. It is possible that being allowed only one alternative will force the physician to make a more careful diagnostic evaluation. However, when the second possible diagnoses were also taken into account, the accuracy rose by only 5–10%. Thus, the possibility of making an alternative diagnosis cannot solely explain the lack of improvement on introduction of the structured schedule. Another possible reason may be that even the introduction of the baseline registration schedule in itself increased the performance, but the design of the present study did not allow determining this effect.

In the present study, the spectrum of diagnoses possible according to the schedule was wide and the physicians had 30 defined diagnoses to choose from. Furthermore, patients who did not fit in any category were classified as “other diagnosis.” In several reports, fewer diagnoses (10 to 19) were used [1, 2, 6, 8, 11, 15, 18]. In an attempt to compare the overall rate of correct diagnoses on admission with that in another report, we analysed the paper by Bjerregaard et al. [15], who used 10 diagnoses: acute appendicitis, acute cholecystitis, diverticular disease, acute intestinal obstruction, NSAP, acute pancreatitis, perforated ulcer, acute salpingitis, ureteric stone, and “other diseases.” The diagnostic performance of the admitting physicians in Copenhagen was 55.1% (*n* = 623). With the same setting of diagnoses, our overall diagnostic performance rate was 54% (*n* = 2062). This comparison and comparisons with the diagnostic performance in other reports [1, 2, 6, 8, 12, 13, 15–18, 20] confirm that the predictive value of the preliminary diagnosis obtained in the present study is well in parity with that of other settings studied, including larger university hospitals.

Furthermore, there was no difference in diagnostic performance in relation to the medical experience of the attending physician. One possible reason could be the well-functioning logistic routines, including frequent communication between the junior doctors at the emergency department and the senior surgeon responsible. It seems that it is the total formal competence that is determining for the diagnostic accuracy, and this is a fact that should not be forgotten in discussions concerning the level of competence of attending physicians at emergency departments [26, 27]. A lower sensitivity of the preliminary diagnosis for women is probably a true picture, but might to some extent depend on the attempt to do the majority of gynaecological investigations during the day-time.

The patient's time of arrival at the emergency department and the possible risk of decreased diagnostic performance during the night-time are often discussed among surgeons. The reasons for such a proposed risk includes a lower degree of education among attending physicians during on-call time, and limited access to advanced diagnostic tools such as radiological imaging and complicated laboratory tests. In this study, however, the diagnostic performance was independent of the time of arrival at the emergency department. One reason obvious from the present data is the lack of correlation between the formal competence of the attending physician and the diagnostic performance.

At many small to medium sized hospitals in Sweden and in other countries with large rural areas, a majority of patients at the emergency department are treated by a nonspecialist physician. Although the use of imaging and advanced laboratory analyses has increased during the past years in the evaluation of emergency abdominal pain, the disposability of such resources is still limited during on-call time at many rural settings. Thus, evaluation of emergency abdominal pain still relies on careful clinical assessment and evaluation of routine laboratory results. At larger hospitals, routine CT scan has been evaluated with diverging results [22, 23]. Immediate availiability of ultrasonograhy [24] in specialised units may also improve the diagnostic accuracy. Furthermore, the use of laparocopy [25], which has the advantage of providing a therapeutic option, has increased the paste years and is mostly available also in rural settings.

One reason for fairly high admission frequency in this study might be that many patients in the catchment area live far, up to 250 kilometres, from the hospital, which does not allow for repeated outpatient assessment shown to be effective in reducing admissions for inpatient care [29].

## 5. Conclusions

A structured schedule for history, symptoms, clinical signs, and results of laboratory examinations did not improve the diagnostic performance in the emergency department as observed by the analyses used in this study. Furthermore, the diagnostic performance was independent of the formal competence of the attending physician as well as of time of arrival of the patient at the emergency department. The diagnostic performance was significantly lower for female patients.

## Figures and Tables

**Table 1:** Category of attending physician at the emergency department and the proportion of correct diagnoses.

	Diagnoses *n*	Proportion diagnoses %	Correct diagnoses *n*	Proportion of correct diagnoses %
Locum	479	16	277	58
Pre-registrar	1409	46	759	54
Senior house officer	811	26	443	55
Specialist/consultant	40	1.3	18	45
General physician (hospital)	195	6.3	103	53
General physician (primary health care)	139	4.5	71	51

Proportion of correct diagnoses between preliminary diagnosis as decided by the physician responsible on admission and final diagnosis after at least one year of follow-up.

**Table 2:** Basic data concerning patients included during the baseline (1996–1997) and study periods (1997–2000).

	Baseline period (hospital)	Study period (hospital)	Study period (primary health care)
Patients included (*n*)	881	3099	238
From the catchment area (*n*)	881	2763	216
Eligible for definitive diagnosis (*n*)	790	2851	222
Age: mean (quartile range) years	46 (24)	44 (44)	40 (44)
Proportion of women (*n*)	419 (53%)	1563 (55%)	128 (58%)

SD: standard deviation.

**Table 3:** Results of validity check of the registration into the database.

Parameter	Errors	
	*n*	%
Time of arrival	1	0.3
Competence of physician	0	0
Temperature	0	0
CRP	3	1
Hospitalised (yes/no)	2	0.7
Surgery (yes/no)	3	1
Time of surgery	0	0
Day of discharge	1	0.3
Diagnosis at discharge	4	1.3

CRP: C-reactive protein.

**Table 4:** Diagnoses on admission (preliminary diagnoses) and after at least one year of follow-up (final diagnoses).

Diagnosis	Hospital *n* = 2851 (preliminary)		Hospital *n* = 2851 (final)		Primary health care, *n* = 222 (preliminary)		Primary health care, *n* = 222 (final)	
	*n*	%	*n*	%	*n*	%	*n*	%
NSAP	641	22	1058	37	52	23	84	38
Gastroenteritis	94	3	64	2.2	14	6.3	4	1.8
Constipation	208	7	130	4.6	13	6	10	4.5
Appendicitis-unspecified	446	16	—	—	36	16	—	—
Appendicitis-phlegmonous	—	—	110	4	—	—	7	3
Appendicitis-gangrenous	—	—	98	3.4	—	—	4	1.8
Appendicitis-perforated	—	—	69	2.4	—	—	7	3
Cholecystitis without perforation	123	4	97	3.4	9	4	6	2.7
Cholecystitis-perforated	—	—	3	0.1	—	—	—	—
Biliary stone pains	287	10	208	7	14	6.3	16	7
Colon-diverticulitis without perforation	161	6	123	4.3	14	6.3	11	5
Colon-diverticulitis-perforated	—	—	11	0.4	—	—	—	—
Obstruction of small intestine without strangulation	80	3	69	2.4	4	2	5	2.3
Obstruction of small intestine with strangulation	2	0.1	9	0.3	—	—	1	0.4
Obstruction of colon	27	1	14	0.5	7	3	3	1.4
Dyspepsia	84	3	60	2	7	3	7	3
Gastric/duodenal ulcer	53	2	26	1	7	3	2	0.9
Gastric/duodenal ulcer-perforated	14	0.5	8	0.3	1	0.5	1	0.4
Acute pancreatitis	70	2.5	92	3.2	3	1.5	2	0.9
Urinary tract infection	106	4	74	2.6	6	3	6	2.7
Urinary tract stone	181	6	107	4	10	4.5	8	4
Urinary tract obstruction	8	0.3	10	0.3	1	0.5	1	0.4
Incarcerated groin hernia	16	0.6	22	1	3	1.5	2	0.9
Incarcerated umbilical hernia	4	0.1	4	0.1	—	—	—	—
Incarcerated incisional hernia	4	0.1	5	0.2	—	—	—	—
Abdominal malignancy	22	0.8	63	2.2	1	0.5	3	1.4
Invagination	2	0.1	1	0.1	1	0.5	—	—
Aortic aneurysm	14	0.5	12	0.4	—	—	1	0.4
Occlusion of mesenteric artery	6	0.2	4	0.1	—	—	—	—
Gynaecological complaint	70	2.5	101	3.5	14	6.3	17	8
Other	128	4.5	199	7	5	2.2	14	6

NSAP: nonspecific abdominal pain.
